# POST-STROKE RISK STRATIFICATION IN PRIMARY CARE: IMPLICATIONS FOR OCCUPATIONAL AND PREVENTIVE MEDICINE

**DOI:** 10.13075/ijomeh.1896.02655

**Published:** 2025

**Authors:** Piotr Gutknecht, Bartosz G. Trzeciak, Marek Tałałaj, Walenty Nyka, Krzysztof Dziewiatowski, Grzegorz Wochyń, Dariusz Świetlik, Janusz Siebert

**Affiliations:** 1 Medical University of Gdańsk, Department of Family Medicine, Gdańsk, Poland; 2 Centre of Postgraduate Medical Education, Department of Orthopedics, Pediatric Orthopedics and Traumatology, Warsaw, Poland; 3 Medical University of Gdańsk, Department of Neurology, Gdańsk, Poland; 4 7th Navy Hospital, Department of Neurology, Gdańsk, Poland; 5 Medical University of Gdańsk, Division of Biostatistics and Neural Networks, Gdańsk, Poland

**Keywords:** primary care, hypertension, risk stratification, cardiovascular risk factors, atrial fibrillation, brain stroke

## Abstract

**Objectives:**

Acute ischemic stroke stands as a significant contributor to high disability and mortality rates. Patient after stroke require vigilant supervision from general practitioners. Cardiovascular prevention emerges as a critical aspect. Physicians play a vital role in managing post-acute care and preventing secondary complications in patients with stroke after discharge. The aim of the study was to characterize and evaluate the cerebrovascular risk factors for stroke in patients under the care of general practitioners.

**Material and Methods:**

Data were collected from 277 patients after ischemic brain stroke under general practitioner care. Baseline demographic and clinical characteristics were gathered for all study participants.

**Results:**

Gender distribution among the study cohort was 143 females (51.6%) aged mean (M) ± standard deviation (SD) 76.4±11.8 years and 134 males (48.4%), aged 78.5±11.9 years. Hypertension emerged as the most prevalent risk factor, affecting 75.8% of participants. Ischemic heart disease, lipid disorders, and atrial fibrillation, observed in 30.32%, 30.7%, and 29.6% of patients respectively. Diabetes mellitus was present in 23.1% of the cohort. The body weight was M±SD 76.9±16 kg, with BMI 27.6±6.6 kg/m^2^. The presence of atrial fibrillation and chronic kidney disease showed statistically significant differences between survival and death groups. Statistically significant differences were observed in diastolic blood pressure in women vs. men (p = 0.0383). Regarding stroke severity, women presented with more severe symptoms (National Institutes of Health Stroke Scale score M = 10.5 vs. 8.3, p = 0.0163) and poorer functional outcomes in modified Rankin Scale (M = 3.3 vs. 2.8, p = 0.0062), a higher prevalence of hypertension and atrial fibrillation among women compared to men (35.0% vs. 17.5%, p = 0.0045).

**Conclusions:**

The authors' findings highlight the necessity for sex-specific approaches in stroke management, particularly considering the impact of comorbid conditions such as hypertension and atrial fibrillation on stroke outcomes. Despite the availability of general guidelines, it would be valuable to develop specific guidelines for general practitioners on ischemic stroke risk factors.

## Highlights

- Hypertension is the most prevalent cardiovascular risk factor post-stroke.
- Women had more severe strokes and worse functional outcomes than men.
- Atrial fibrillation and chronic kidney disease were associated with higher mortality.
- Stroke survivors require tailored, sex-specific prevention strategies.

## INTRODUCTION

Acute ischemic stroke (AIS) is a significant contributor to global disability and mortality, affecting approx. 795 000 individuals each year with both new and recurrent cases [1]. Between 1996 and 2006, there was a notable 33.5% reduction in stroke mortality, accompanied by an 18.4% decline in stroke-related deaths [2]. In Poland, the incidence rate is estimated at an average of 150/100 000 inhabitants, with men about 19% more likely to experience stroke, although this trend tends to even out in older age groups [3]. According to some studies conducted in Poland, an 8% decrease in hospitalisation rates from 169/100 000 population in 2009 to 157/100 000 in 2013 was noted [3]. Recently, more strokes are being observed in young people. It is estimated that about 10% of patients are <50 years old. Among the causes of strokes in young people are work-related factors, including long working hours and occupational noise [4,5]. Stroke is a preventable and treatable disease, the burden of which may be reduced. The healthcare burden imposed by stroke is profound, with substantial financial implications due to high annual healthcare costs. The pathophysiology of stroke is highly complex, involving mechanisms such as excitotoxicity, inflammation, oxidative stress, ionic imbalances, apoptosis, angiogenesis, and neuroprotection.

Stroke is a leading cause of disability-adjusted life years globally. General practitioners (GPs) play a crucial role in optimising secondary prevention after stroke by addressing cerebrovascular risk factors and ensuring comprehensive management in line with clinical practice guidelines. In primary healthcare settings, effective prevention remains paramount in mitigating the impact of stroke. The SCORE algorithm is a tool for assessing 10-year risk of cardiovascular death [6]. Nearly 90% of stroke cases are attributable to various risk factors including hypertension (HT), atrial fibrillation (AF), lack of physical activity, dyslipidaemia, poor diet, psychosocial factors, smoking, cardiac conditions, excessive alcohol consumption, and diabetes mellitus (DM) [1]. Modifiable risk factors such as HT, smoking, DM, cardiac conditions, poor diet, lack of physical activity, and obesity are well-documented. While these factors often exert independent effects, understanding their interactions is crucial for predicting overall risk and designing effective risk modification programmes.

Stroke is considered a disease caused by long-term exposure to lifestyle-related risk factors. Modifiable risk factors need to be identified and the efficacy of risk reduction strategies must be demonstrated in order to reduce the stroke burden. Despite existing studies, including those investigating clinical characteristics and topography of ischemic stroke, few have specifically addressed risk factors and their correlation with neurological outcomes in patients under GPs supervision [7,8].

The aim of the study was to characterize and evaluate the risk factors for stroke in patients under the care of GPs.

## MATERIAL AND METHODS

A group of consecutive 277 patients under GP supervision was admitted to 2 neurology departments for first-ever AIS in 2018 and enrolled in this study. The diagnosis of ischemic stroke was defined as an onset of a new and sudden focal neurological deficit, with symptoms persisting for >24 h, confirmed by appropriately located ischemic lesions on head computed tomography (CT) or magnetic resonance imaging (MRI). Exclusion criteria encompassed conditions such as aortic regurgitation, intra-aortic balloon pump, pleural effusion or pulmonary oedema, severe obesity or malnutrition. The data were collected over a 1-year period from hospital medical records and GPs documentation. Collaboration with GPs enabled a 6 months follow-up of post-stroke patients in the primary healthcare setting.

Various demographic and anthropometric details were assessed alongside clinical parameters, providing insights into neurological and functional aspects. Management guidelines and lifestyle recommendations were provided to patients during the acute phase of stroke. All definitions of risk factors were based on international guidelines of European Society of Cardiology (ESC) and the European Society of Hypertension (ESH) criteria.

Baseline demographic and clinical data were meticulously collected for all participants, encompassing cardiovascular risk factors, stroke aetiology classified by the Trial of Org 10172 in Acute Stroke Treatment (TOAST) criteria, clinical syndrome according to the Oxfordshire Community Stroke Project (OCSP) criteria, National Institutes of Health Stroke Scale (NIHSS) score on admission, Glasgow Coma Scale (GCS) on admission, thrombolytic treatment, and modified Rankin Scale (mRS) score at discharge. The study was approved by the Ethical Committee of the Medical University of Gdańsk, Poland (No. NKBBN/64-451/2015).

Statistical analyses were conducted with the StatSoft Inc. statistical package (2014), Statistica v. 12.0, and Microsoft Excel spreadsheet. Quantitative variables were characterised using descriptive statistics including mean (M), standard deviation (SD), median (Me), min. and max values (range), and 95% confidence intervals (CI), while qualitative variables were presented as counts and percentages.

Normality of distribution for quantitative variables was assessed using the Shapiro-Wilk test, and the Levene test (Brown-Forsythe test) was employed to evaluate the hypothesis of equal variances. Differences between groups for continuous variables were assessed using the Student's t-test or the Mann-Whitney U test, depending on data characteristics. Chi-square independence tests were applied for categorical variables, with a significance level of p = 0.05.

## RESULTS

The study provided a comprehensive analysis of medical conditions and lifestyle factors among participants, revealing a balanced gender distribution with 143 females (51.6%) and 134 males (48.4%), with age M±SD 78.5±11.9 years for females and 76.4±11.8 years for males. Hypertension emerged as the most prevalent risk factor (75.5%), followed by ischemic heart disease (32.3%), lipid disorders (32.1%), and AF (29.6%). Diabetes mellitus was present in 23.8% of participants. In the studied group body weight is M±SD 76.9±16.0 kg, the average height is M±SD 167.3±9.6 cm, and the average BMI is M±SD 27.6±6.6 kg/m^2^. The percentage of respondents who completed primary education is 27.3%, secondary education 55.6%, and higher education 17.1%. The percentage of those employed is 20.7%, while retirees constitute 79.3%.

The percentage of stroke in the group of women and men are 51.6% vs. 48.4%, respectively. No statistically significant difference was found (p = 0.5887). No statistically significant age difference between genders was observed (p = 0.1477). The BMI in the group of women and men is M±SD 27.3±5.7 kg/m^2^ vs. 27.8±7.5 kg/m^2^ and did not differ significantly (p = 0.5). In the male group, diastolic blood pressure was significantly higher than in women (M±SD 79.6±10.8 mm Hg vs. 76.5±13.3 mm Hg, p = 0.0383). The systolic blood pressure in the group of women and men is M±SD 142.8±25.9 mm Hg vs. 140.9±18.8 mm Hg. No statistically significant systolic blood pressure difference between genders was observed (Table 1).

**Table 1.** Clinical data of patients under the supervision of a general practitioner, admitted to 2 neurology departments for a first-ever acute ischemic stroke, Poland, 2018

Variable	Participants (N = 277)		p
	female (N = 143)	male (N = 134)	
Strokes [n (%)]	143 (51.6)	134 (48.4)	0.5887
Age [years]			0.1477
M±SD	78.5±11.9	76.4±11.8	
range	33.0–98.0	40.0–98.0	
Me (IQR)	82.0 (18.0)	76.5 (20.0)	
BMI [kg/m^2^]			0.5098
M±SD	27.3±5.7	27.8±7.5	
range	17.3–62.5	18.3–98.5	
Me (IQR)	26.8 (7.0)	26.9 (5.2)	
Blood pressure [mm Hg]			
diastolic			0.0383
M±SD	76.5±13.3	79.6±10.8	
range	44.5–137.3	56.4–105.9	
Me (IQR)	75.5 (15.1)	79.0 (12.4)	
systolic			0.4856
M±SD	142.8±25.9	140.9±18.8	
range	88.8–236.0	88.9–203.8	
Me (IQR)	137.3 (31.7)	139.3 (29.5)	

Women had higher high-density lipoprotein than man (M±SD 56.7±25.6 mg/dl vs. 46.6±20.7 mg/dl, p = 0.000). A smoking exposure was no significantly higher in men group (Me = 40 packs/year vs. Me = 30 packs/year, p = 0.1085). These findings suggest that while glucose levels remain similar between sexes, lipid metabolism and smoking exposure exhibit notable differences, which may impact long-term vascular risk (Table 2).

**Table 2.** Characteristics of glucose levels, total cholesterol, low-density lipoprotein (LDL), high-density lipoprotein (HDL), triglycerides and annual cigarette consumption in patients under the supervision of a general practitioner, admitted to 2 neurology departments for a first-ever acute ischemic stroke, Poland, 2018

Variable	Participants (N = 277)			p^a^
	total	women (N = 143)	men (N = 134)	
Glucose [mg/dl]				
day 1				0.5957
M±SD	160.4±51.0	160.7±42.7	160.0±59.3	
range	73.0–286.0	93.0–263.0	73.0–286.0	
Me (IRQ)	150.0 (69.0)	167.0 (66.0)	145.0 (70.0)	
day 5–7				0.2677
M±SD	129.2±51.3	128.2±35.0	130.3±64.9	
range	75.0–381.0	83.0–226.0	75.0–381.0	
Me (IRQ)	118.0 (39.0)	120.0 (40.0)	115.0 (24.0)	
Cholesterol [mg/dl]				
total				0.0850
M±SD	179.5±47.3	187.7±51.9	171.4±41.0	
range	89.0–370.0	114.0–370.0	89.0–289.0	
Me (IRQ)	178.0 (50.9)	181.0 (44.0)	167.0 (54.0)	
LDL				0.8813
M±SD	111.0±45.6	112.8±46.9	109.3±44.5	
range	32.0–350.0	32.0–290.0	34.0–350.0	
Me (IRQ)	105.6 (46.1)	105.6 (46.8)	106.2 (47.9)	
HDL				0.0003
M±SD	51.5±23.7	56.7±25.6	46.6±20.7	
range	21.0–195.8	28.0–195.8	21.0–182.0	
Me (IRQ)	47.0 (19.0)	52.6 (24.0)	42.0 (19.0)	
Triglycerides [mg/dl]				0.5753
M±SD	119.2±58.0	116.3±56.0	121.9±60.2	
range	45.0–382.0	45.0–371.0	46.0–382.0	
Me (IRQ)	113.0 (59.0)	109.0 (65.0)	118.0 (58.0)	
Cigarettes packs [n/year]				0.1085
M±SD	38.1±37.9	28.3±14.3	42.0±43.5	
range	5.0–300.0	5.0–50.0	8.0–300.0	
Me (IRQ)	38.0 (25.0)	30.0 (25.0)	40.0 (28.0)	

a Women vs. men.

Women had significantly higher NIHSS scores than men (Me = 9.0, interquartile range [IQR] 10.0 vs. Me [IQR] = 7.0 [7.0], p = 0.0163), indicating greater stroke severity. Glasgow Coma Scale scores were lower in women (Me [IQR] = 14.0 [2.0] vs. Me [IQR] = 15.0 [1.0], p = 0.0119), suggesting reduced consciousness. Women also had worse functional outcomes, with higher mRS scores (M±SD 3.3±1.3 vs. 2.8±1.3, p = 0.0062) (Table 3).

**Table 3.** Characteristics of patients under the supervision of a general practitioner, admitted to 2 neurology departments for a first-ever acute ischemic stroke, in terms of the modified Rankin Scale (mRS) score, the National Institutes of Health Stroke Scale (NIHSS) score, and the Glasgow Coma Scale (GCS), Poland, 2018

Scale	Participants (N = 277)			p^a^
	total	women (N = 143)	men (N = 134)	
NIHSS				0.0163
M±SD	9.4±6.5	10.5±7.2	8.3±5.6	
range	0.0–33.0	0.0–33.0	0.0–25.0	
Me (IRQ)	8.0 (8.0)	9.0 (10.0)	7.0 (7.0)	
GCS				0.0119
M±SD	13.6±2.3	13.3±2.6	14.1±1.9	
range	4.0–15.0	4.0–15.0	4.0–15.0	
Me (IRQ)	15.0 (1.0)	14.0 (2.0)	15.0 (1.0)	
mRS				0.0062
M±SD	3.1±1.4	3.3±1.3	2.8±1.3	
range	0.0–5.0	0.0–5.0	0.0–5.0	
Me (IRQ)	3.0 (2.0)	3.0 (2.0)	3.0 (2.0)	

a Women vs. men.

Hypertension did not show a statistically significant difference between survival and death groups, indicating the need for further exploration of its impact on stroke outcomes (Table 4).

**Table 4.** The attributes of the patients under the supervision of a general practitioner, admitted to 2 neurology departments for a first-ever acute ischemic stroke, by their comorbidities, Poland, 2018

Variable	Participants (N = 277) [n (%)]		p
	survival (N = 214)	death (N = 63)	
Hypertension			0.6969
no	50 (23.7)	17 (27.4)	
yes			
treated	138 (65.4)	38 (61.3)	
not treated	20 (9.5)	7 (11.3)	
just diagnosed	3 (1.4)	0 (0.0)	
Chronic kidney diseases			0.0315
no	193 (93.7)	51 (85.0)	
yes	13 (6.3)	9 (15.0)	
Diabetes			0.4965
no	159 (77.2)	46 (73.0)	
yes	47 (22.8)	17 (27.0)	
Ischemic heart disease			0.1220
no	139 (70.2)	37 (59.7)	
yes	59 (29.8)	25 (40.3)	
Lipid disorders			0.0991
no	134 (65.4)	46 (76.7)	
yes	71 (34.6)	14 (23.3)	
Atrial fibrillation			0.0015
no	150 (73.9)	32 (52.5)	
yes	53 (26.1)	29 (47.5)	
Atrial fibrillation treated with anticoagulant			0.2504
no	27 (50.9)	18 (64.3)	
yes	26 (49.1)	10 (35.7)	
Cigarettes			0.1744
never	75 (43.1)	22 (57.9)	
active smoking	56 (32.2)	7 (18.4)	
quit >2 years ago	43 (24.7)	9 (23.7)	
Heart failure			0.0571
no	181 (88.3)	48 (78.7)	
yes	24 (11.7)	13 (21.3)	
Veins thrombosis			0.1805
no	196 (97.0)	59 (100.0)	
yes	6 (3.0)	0 (0.0)	

In the survivor group, women had a significantly higher prevalence of HT (82.2% vs. 70.2%, p = 0.0396) and AF (35.0% vs. 17.5%, p = 0.0045) compared to men. Heart failure (HF) was also more frequent in women (16.5% vs. 6.9%, p = 0.0318). In contrast, men had a significantly higher smoking rate (74.2% vs. 38.8%, p < 0.0001). No significant differences were observed between sexes in chronic kidney diseases (CKD), diabetes, ischemic heart disease, lipid disorders, anticoagulant treatment, or venous thrombosis (Table 4).

Comparison of cardiovascular risk factors in stroke non-survivors indicated that only smoking was significantly more common in men (61.1% vs. 25.0%, p = 0.0244). Diabetes was more frequent in men (37.9% vs. 17.6%, p = 0.0706), CKD was more prevalent in women (20.6% vs. 7.7%, p = 0.1657) though not statistically significant (Table 5).

**Table 5.** Comparison of cardiovascular risk factors and comorbidities, divided by gender, in patients under the supervision of a general practitioner, admitted to 2 neurology departments for a first-ever acute ischemic stroke, Poland, 2018

Variable	Participants (N = 277) [n (%)]					
	survival (N = 214)		p^a^	death (N = 63)		p^a^
	women	men		women	men	
Hypertension			0.0396			0.6984
yes	88 (82.2)	73 (70.2)		24 (70.6)	21 (75.0)	
no	19 (17.8)	31 (29.8)		10 (29.4)	7 (25.0)	
Chronic kidney disease			0.3704			0.1657
yes	5 (4.8)	8 (7.8)		7 (20.6)	2 (7.7)	
no	99 (95.2)	94 (92.2)		27 (79.4)	24 (92.3)	
Diabetes			0.2773			0.0706
yes	27 (26.0)	20 (19.6)		6 (17.6)	11 (37.9)	
no	77 (74.0)	82 (80.4)		28 (82.4)	18 (62.1)	
Ischemic heart disease			0.5147			0.1586
yes	31 (32.0)	28 (27.7)		11 (32.4)	14 (50.0)	
no	66 (68.0)	73 (72.3)		23 (67.6)	14 (50.0)	
Lipids disorders			0.9236			0.5654
yes	36 (35.0)	35 (34.3)		7 (20.6)	7 (26.9)	
no	67 (65.0)	67 (65.7)		27 (79.4)	19 (73.1)	
Atrial fibrillation			0.0045			0.9326
yes	35 (35.0)	18 (17.5)		16 (47.1)	13 (48.1)	
no	65 (65.0)	85 (82.5)		18 (52.9)	14 (51.9)	
Atrial fibrillation treated with anticoagulant			0.1006			0.8199
yes	20 (57.1)	6 (33.3)		6 (37.5)	4 (33.3)	
no	15 (42.9)	12 (66.7)		10 (62.5)	8 (66.7)	
Cigarettes			<0.0001			0.0244
smoking	33 (38.8)	66 (74.2)		5 (25.0)	11 (61.1)	
never	52 (61.2)	23 (25.8)		15 (75.0)	7 (38.9)	
Heart failure			0.0318			0.1574
yes	17 (16.5)	7 (6.9)		5 (14.7)	8 (29.6)	
no	86 (83.5)	95 (93.1)		29 (85.3)	19 (70.4)	
Veins thrombosis			0.4072			–
no	97 (96.0)	99 (98.0)		34 (100.0)	25 (100.0)	
yes	4 (4.0)	2 (2.0)		0 (0.0)	0 (0.0)	

a Women vs. men.

The diastolic and systolic blood pressure did not show a statistically significant difference between survival and death groups, indicating the need for further exploration of its impact on stroke outcomes (Table 6).

**Table 6.** Characteristics of the group of patients under the supervision of a general practitioner, admitted to 2 neurology departments for a first-ever acute ischemic stroke, in terms of the blood pressure and mortality, Poland, 2018

Blood pressure	Participants (N = 277)		p
	survival (N = 214)	mortality (N = 63)	
Diastolic [mm Hg]			0.4670
M±SD	77.6±11.4	79.5±14.5	
range	44.5–115.6	56.4–137.3	
Me (IRQ)	76.9 (13.3)	78.9 (17.9)	
Systolic [mm Hg]			0.2358
M±SD	141.0±22.3	145.1±24.1	
range	88.8–236.0	99.2–223.6	
Me (IRQ)	137.3 (29.7)	143.0 (30.8)	

## DISCUSSION

This study provides insights into the characteristics and outcomes of 277 ischemic stroke patients, revealing key factors influencing stroke severity and survival. The mean age of >77 years highlights the relevance of stroke in older populations, with moderate stroke severity as indicated by the NIHSS and GCS scores. Comorbidities such as CKD and AF were strongly associated with higher mortality, emphasizing their role in poor stroke outcomes. Elevated glucose levels and smoking also emerged as significant contributors to stroke risk. However, while blood pressure and lipid levels were slightly higher in patients who died, they were not statistically significant in predicting mortality.

With the rise in life expectancy, AIS, characterised by high morbidity rates, has emerged as a significant public health concern. In a typical primary care practice of 2000 adults, 100 will have a history of stroke, and 5–10 will have a new stroke each year [9]. In Poland, a network of hospital stroke units has been developed since 2002. Most patients with ischemic and recurrent stroke are treated there according to the guidelines of the Polish Neurological Society [3]. Family doctors are a significant element of patients' health promotion. This can reduce the risk of stroke in the long term. Primary care teams also provide the majority of post-stroke care. Notably, the incidence of stroke escalates with age, doubling for each decade after the age of 55 years, as evidenced by age M = 77.5 years [10]. A decline of stroke hospitalisation rates in Poland among the elderly was observed from 2010 to 2019 [3]. The study comprised a higher proportion of female patients in both the recurrent ischemic stroke cohort and the first-ever ischemic stroke cohort.

The risk of stroke increases with age. The first stroke incident at a younger age is mostly related to vascular pathology [3]. While the risk of stroke is comparable between men and women at younger ages, studies suggest a slightly higher relative risk for men in older age groups [11]. It may be due to protective effects of estrogens [12]. However, functional post-stroke prognosis appears to vary between genders, with some studies indicating worse outcomes for women initially, but no significant differences in the long term [11]. Age remains a crucial non-modifiable risk factor and a significant predictor of recurrent strokes. Racial disparities in stroke have also been documented [3]. African Americans have a double risk of experiencing a new stroke compared to individuals of white ethnicity, and they also have higher mortality associated with stroke.

The findings of this study suggest that female stroke patients may have a greater burden of certain risk factors compared to males, contributing to poorer stroke outcomes. Specifically, the higher prevalence of HT in women (82.2% vs. 70.2%, p = 0.0396) and the significantly greater proportion of women with AF (35.0% vs. 17.5%, p = 0.0045) indicate these comorbidities as key contributors to stroke severity. Atrial fibrillation is a well-established risk factor for ischemic stroke and may exacerbate the adverse effects of stroke in women, potentially leading to higher disability rates post-stroke [3].

Additionally, the authors' analysis of sex differences in stroke severity using the NIHSS and mRS suggests that women tend to experience more severe strokes with worse functional outcomes.

The INTERSTROKE study highlighted ten modifiable risk factors accounting for a substantial portion of stroke risk, including HT, smoking, abdominal obesity, diet quality, physical inactivity, DM, binge alcohol consumption, psychosocial stress and depression, cardiac disease, and apolipoprotein B to A1 ratio [13]. However, the association between blood pressure variability and stroke risk remains debatable, with conflicting findings among studies such as the Cardiovascular Health Study [14]. In both recurrent and first-ever ischemic stroke cases, HT stands out as the most notable modifiable risk factor, followed by dyslipidaemia, DM, smoking, and hyperuricaemia. These findings align with the outcomes observed in prior research [12]. Even among individuals who do not meet the criteria for HT, elevated blood pressure levels are associated with an increased risk of stroke. Despite its high prevalence, HT did not show a statistically significant difference between survival and death groups, indicating the need for further exploration of its impact on stroke outcomes (Table 5). For instance, British researchers, analysing data from 4 randomised controlled trials involving patients with HT, prior stroke, or prior transient ischemic attack, discovered that variability in blood pressure readings (2–10 measurements over approx. 2 years) is a risk factor for stroke, regardless of the mean blood pressure level [15]. Despite available studies, only few have investigated the relationship between risk factors and neurological outcomes in recurrent ischemic stroke patients. Patients presenting with multiple risk factors often pose challenges in managing their conditions, making them more susceptible to recurrent strokes. In Arboix et al. study [16] HT was the primary cardiovascular risk factor specifically for lacunar infarcts and atherothrombotic infarctions, both of which are ischemic strokes linked to small- and large-vessel disease. These stroke subtypes were significantly more prevalent in the hypertensive group, which was characterized by older age, a higher incidence of prior cerebral infarctions, elevated rates of hyperlipidemia, acute stroke onset, lacunar syndromes, and infarcts located in the pons.

In the authors' study there was variation in blood pressure between survivors and non-survivors, none of the differences are statistically significant. This highlights the importance of considering a multifactorial approach when predicting mortality in stroke patients, with BP being only 1 of many parameters to monitor. Furthermore, while blood pressure control is essential in managing stroke patients to prevent complications like hemorrhagic transformation or worsening ischemia, these results imply that BP levels may not directly correlate with mortality. The authors' data pointed out the differences in blood pressure (particularly diastolic blood pressure) between men and women post-stroke (M = 76.5 mm Hg vs. M = 79.6 mm Hg, p = 0.0383) may indicate sex-specific vascular responses. Higher diastolic blood pressure in men could suggest a distinct pathophysiological response to stroke, necessitating tailored blood pressure management strategies.

Dyslipidaemia, another prevalent risk factor, underscores the complex interplay between lipid levels and the risk of stroke, with varying associations observed based on cholesterol and stroke subtypes. In Poland, nearly 20 million individuals have hypercholesterolaemia, with the majority of them unaware of their condition. Consequently, only approx. 5% of patients with familial hypercholesterolaemia have been diagnosed. This lack of awareness also contributes to the rarity of diagnoses for other rare cholesterol metabolism disorders in Poland [17].

In the authors' study, almost 47% of patients presented lipid disorders. Dyslipidemia was evident, with elevated low-density lipoprotein (LDL) and total cholesterol levels alongside borderline low high-density lipoprotein (HDL) levels in some patients, indicating a need for lipid-lowering interventions to prevent secondary vascular events.

There exists a strong and direct correlation between total cholesterol, LDL, and ischemic stroke. Oxidized cholesterol, particularly LDL, instigates inflammation and contributes to the formation of plaque on the inner walls of blood vessels, thus obstructing arterial blood flow [18]. While some studies suggest conflicting and contrasting findings on the association between dyslipidaemia and the risk of ischemic and haemorrhagic stroke, statin use seems to decrease the risk of total and ischemic stroke without a clear increase in the risk of haemorrhagic stroke in the general population [17]. Vitturi et al. [19] found that a comprehensive analysis of the lipid panel enables an assessment of the prognosis for patients who have experienced a stroke. Elevated LDL-C levels >70 mg/dl were identified as an independent predictor of an increased risk of recurrent stroke and poorer functional outcomes [18].

Topor-Madry et al. [20] described that the estimated prevalence of DM in Poland is 6.97%. The total number of people with DM in Poland was 2.68 million. In the authors' study the mean glucose level on admission was elevated. Reduction observed in consecutive days may indicate a decline in acute stress-related hyperglycemia as recovery progresses.

Diabetic patients who experience a stroke typically exhibit a younger age profile and have a higher prevalence of other risk factors associated with stroke. Glycated haemoglobin levels were also found to be a prognostic indicator, with poorer outcomes associated with higher levels [20]. Effective management strategies combining lifestyle modifications and pharmacotherapy are crucial in reducing stroke risks in diabetic individuals.

Atrial fibrillation was prevalent in a significant proportion of the study group, consistent with its recognition as a major risk factor for stroke, especially in women compare to the men without anticoagulation medication. Yiin et al. [21] pointed out that incident stroke related to AF has nearly tripled in the past 3 decades. The prevalence of AF in the Polish population ≥65 years was estimated at 19.2% in 2022 [22]. The impact of AF on stroke prognosis extends beyond neurological deficits and age, with females experiencing higher stroke risks than males [22].

Tobacco smoking, a well-established risk factor for cardiovascular diseases, was prevalent among this study population, necessitating targeted interventions for cessation to mitigate stroke risks. It is likely that stroke has a dose-dependent relationship with smoking [23]. Cigarette smoking continues to represent a significant risk factor for stroke, nearly doubling the risk, with a dose-response relationship observed between pack-years of smoking and the risk of stroke [13]. The authors' results emphasize the role of smoking as a significant modifiable risk factor, with men demonstrating a substantially higher smoking prevalence (74.2% vs. 38.8%, p < 0.0001). While smoking is a well-documented stroke risk factor, this study suggests that its impact may differ by sex, warranting consideration in stroke prevention strategies.

Renal failure emerged as a potent risk factor for stroke, with patients facing significantly higher stroke risks, particularly those undergoing dialysis. The relationship between survivors and non-survivors with CKD in the authors' study was considered to be statistically significant (p < 0.0315). Renal failure is a significant risk factor for stroke, a leading contributor to both morbidity and mortality globally.

Patients with CKD, particularly those undergoing dialysis, face a markedly elevated risk of stroke, 5–30 times higher compared to the general population [24].

Heart failure, although not statistically significant in the authors' study, remains a complex prognostic factor, with stroke mechanisms varying based on HF subtype. There is growing evidence suggesting that HF independently increases the risk of stroke, irrespective of AF, primarily due to thromboembolism. However, earlier studies have not demonstrated the efficacy of warfarin in HF patients without AF, as the benefits of stroke prevention were offset by a higher occurrence of major bleeding events [25].

Primary care often faces challenges in meeting treatment targets, especially with non-compliant patients. Many factors influencing outcomes extend beyond the scope of primary care providers. One year post-stroke, while 97% of eligible patients continue antiplatelet therapy, only 50–70% achieve blood pressure <140/90 mm Hg. Furthermore, 79% remain on statin therapy, 84% sustain a non-smoking status, and 48% adhere to exercise recommendations. However, merely 17% attain a healthy weight [9].

Despite a small sample size, the patient selection criteria were stringent. This allowed for the collection of targeted data that enhances the reliability of the results. The authors' findings correspond with studies in larger populations. Observed trends are clinically significant and have practical implications for patient management. Although the study is limited by hospital-based design and relatively small sample size, which restricts generalizability, it highlights clinically important gender-specific differences in comorbidities and outcomes. These observations may support the need for tailored preventive strategies in primary care and occupational health practice. Another limitation is potential unmeasured confounding factors such as the patients' compliance with treatment, the severity of comorbidities, or variations in the quality of post-stroke care. Lack of detailed analysis on the topography of the infarcts could influence outcomes. As reported in previous studies understanding detailed mechanisms underlying ischemic stroke mainly in its different stroke subtypes may lead to the development of effective therapeutic strategies [26].

Future research should focus on expanding the study population to include a larger, more diverse cohort, which would allow for a deeper analysis of the associations between specific comorbidities and stroke outcomes. Additionally, investigating the role of stroke subtypes and lesion topography in determining functional recovery and mortality would offer valuable insights. A more comprehensive follow-up data, including the exact causes of death, would be beneficial for a more accurate assessment of factors influencing long-term survival in ischemic stroke patients.

## CONCLUSIONS

The authors' findings highlight the necessity for sex-specific approaches in stroke management, particularly considering the impact of comorbid conditions such as HT and AF on stroke outcomes. Despite the availability of general guidelines, it would be valuable to develop specific guidelines for GP on ischemic stroke risk factors.
